# A Memoir on Uretro-Plastie

**Published:** 1845-10

**Authors:** 


*A Memoir on Uretro-Plastie.* By M. Segalas.—M. Segalas, in a letter addressed to M. Dieffenbach, in 1840, stated his opinion, that the attempts at reparation of a breach of substance of the urethra had hitherto failed in consequence of the contact of the urine with the parts concerned not having been prevented; and, at the same time, detailed the particulars of a case in which, this latter object being attained by making a preliminary incision in the perineum, and introducing a catheter through it into the bladder, a complete cure resulted. A second case was similarly treated by M. Ricord, and a third forms the subject of the present communication.

This patient, aged 30, exhibited a large loss of the spongy portion of the urethra, the consequence of gangrene supervening upon a ligature he had attached to the penis when a child. The urine and seminal fluid passed entirely by this aperture, which was an inch in length, and occupied the entire thickness of the urethra, a deep transverse cicatrix being observed also to surround the corpus cavernosum. The portion of the urethra anterior to the loss of substance was much narrowed, but easily admitted a probe, and when the patient approached the two orifices of the defective portion he could force urine through it. The operation was divided into three stages; in the first, the prepuce, which was phymotic, was divided, in order to relax the parts afterwards to be operated upon. Six days after, a free opening was made into the membranous portion of the urethra, and a gum-elastic catheter introduced. The urine thus being prevented coming in contact with the canal, and the parts being in a tranquil state, the operation was completed, three weeks after the first incision of the prepuce, by dividing the edges of the defective portions of urethra and bringing them into contact over a bougie by means of the twisted suture. The wound healed kindly, except at one minute point, which remained fistulous for a very long period, notwithstanding the application of various cauteries, &c. It, however, eventually completely healed, though not until its orifice had been incised. The operation was performed the 18th August, and the catheter removed from the perineum the 26th November. Dieffenbach had objected to this operation, that the wound made in the perineum might itself remain fistulous. Such has not been the case in the other cases reported, and in the present one, it closed in a few days after the removal of the instrument. Although perfectly able to evacuate the bladder by the urethra, the patient was recommended to do so for a considerable time by means of a catheter. Throughout the treatment of the case neither fever or irritation manifested itself.—*Ibid.*

